# Comparative Evaluation of Locally Administered 2% Gel Fabricated from Lemongrass Polymer and 10% Doxycycline Hyclate Gel as an Adjunct to Scaling and Root Planing in the Treatment of Chronic Periodontitis—A Randomized Controlled Trial

**DOI:** 10.3390/polym14142766

**Published:** 2022-07-06

**Authors:** Pooja Mittal, Shankar T. Gokhale, Shiva Manjunath, Saad M. Al-Qahtani, Mohammad Al. Magbol, Raghavendra Reddy Nagate, Shreyas Tikare, Saurabh Chaturvedi, Ashish Agarwal, Vatsala Venkataram

**Affiliations:** 1 Department of Periodontology, Dental College Azamgarh, Azamgarh 276128, Uttar Pradesh, India; drmittalpooja@gmail.com; 2 Department of Periodontics and Community Dental Sciences, College of Dentistry, King Khalid University, Abha 61421, Saudi Arabia; sgokhale@kku.edu.sa (S.T.G.); saad71@outlook.in (S.M.A.-Q.); moama1957@outlook.com (M.A.M.); rnagati@kku.edu.sa (R.R.N.); tikane@kku.edu.sa (S.T.); 3 Department of Periodontics, Institute of Dental Sciences, Bareilly 243006, Uttar Pradesh, India; drmanju75@rediffmail.com; 4 Department of Prosthetic Dentistry, College of Dentistry, King Khalid University, Abha 61421, Saudi Arabia; 5 Community Health Center, Puranpur, Pilibhit 262121, Uttar Pradesh, India; drashish.aag@gmail.com; 6 Department of Pedodontics & Preventive Dentistry, KVG Dental College & Hospital, Sullia 574327, Karnataka, India; dr.vatsala@gmail.com

**Keywords:** cymbopogon citratus, local drug delivery, non-surgical therapy, periodontal pathogens

## Abstract

Background: Extracts of medicinal plant like lemongrass offer a new choice for optional antimicrobial therapy against various oral microorganisms. The objective of this study was to assess, verify, and compare the antimicrobial effectiveness of locally administered 2% lemongrass gel and 10% doxycycline hyclate gel as an adjunct to scaling and root planing (SRP) in treating chronic periodontitis. Method: This is a double-blind parallel arm randomized controlled study. Forty subjects were randomly divided into Group A and B for 2% lemongrass gel and 10% doxycycline hyclate gel, respectively. The clinical assessments of Gingival Index (GI), Plaque Index (PI), Probing Pocket Depth (PPD), and Clinical Attachment Level (CAL) together with microbial colony counts for *Porphyromonas gingivalis*, *Actinomyces naeslundii,* and *Prevotella intermedia* were done at baseline, 1st month, and 3rd month follow-ups. Results: The results showed there was a significant reduction in the mean scores of GI, PPD, and CAL clinical indices from baseline to the 1st and 3rd month follow-ups in both the 2% lemongrass gel and 10% doxycycline gel groups (*p* < 0.05). Similarly, there was significant reduction in mean CFU scores for all periodontal pathogens from baseline to 1st and 3rd month follow-ups in both the 2% lemongrass gel and 10% doxycycline gel groups (*p* < 0.05). Conclusions: It could be concluded that the local delivery of 2% lemongrass gel as an adjunct to scaling and root planing is effective and comparable to 10% doxycycline gel in the treatment of chronic periodontitis.

## 1. Introduction

The applications of polymers in dentistry are increasing day by day. The irreplaceable merits of different classes of natural polymers over synthetic polymers as drug carriers still urge researchers to depend solely on biomaterials. These advantages include hydrophilicity, biocompatibility, non-immunogenicity, non-toxicity, non-toxic degradation products, antimicrobial and antioxidant capacities, and high stability for tissue engineering [1,2,3]. Also, from an environmental perspective, the worldwide demand for eco-friendly products has witnessed sustainable growth for accommodating green technology advancements. Hence, industrial channels are searching for more reliable products to gain patients’ satisfaction and ease their commercialization [4,5,6,7,8].

One such field is pain relief in dentistry. The use of polymers in dentistry has evolved from simple reinforcement for dental materials to application in healing and pain relief. Periodontitis is defined as ‘an inflammatory disease of the supporting tissues of the teeth caused by specific microorganisms or groups of specific microorganisms, resulting in progressive destruction of the periodontal ligament and alveolar bone with increased probing depth formation, recession, or both’ [1]. The most prevalent periodontal disease is chronic periodontitis, which is caused by the deposition of bacterial plaque over time. The therapy for gingivitis and periodontitis has evolved quite differently in these two areas, depending on the advancements made in the area of drug and polymer development and changes in patient populations and needs. The advancements that are most pertinent to polymers dealt with (1) their use as carriers for the controlled delivery of bioactive agents, particularly antimicrobials (2) their use in conjunction with tissue regeneration and healing and (3) factors pertinent to the effectiveness of implemented therapies [2,9,10,11,12,13,14].

Cymbopogon citratus, Stapf, or lemongrass, is a naturally available flavonoid and contains cellulose as a natural polymer. This lemongrass polymer is working towards the quest for the development of a biodegradable natural polymer that has anti-bacterial, anti-filarial, anti-fungal, anti-inflammatory, and antioxidant properties [15]. Lemongrass has active phenol and flavonoid substances which, at concentrations below 2%, have shown to have bacteriostatic properties against several microorganisms [16]. In an in vitro study, lemongrass essential oil was effective against periodontal pathogens like *Actinomyces naeslundii* and *Porphyromonas gingivalis* and also a majority of clinical-isolate groups, including tetracycline hydrochloride-resistant strains [17]. Mouth rinse with active Cymbopogon citratus essential oil as an adjunct to SRP has shown to be effective in reducing the severity of gingivitis [18,19]. The use of lemongrass essential oil as a local drug delivery (LDD) in chronic periodontitis is limited.

Non-surgical mechanical periodontal therapies such as *scaling* and *root planing* (SRP), and in some cases surgical periodontal therapy with access flaps, have been archived widely in the literature to hamper the progression of tissue destruction in periodontal disease [2]. Mechanical therapy, however, may not always reduce or eliminate the anaerobic infection at the base of the pocket, within the gingival tissues, and in areas inaccessible to periodontal instruments [3]. The reduction or elimination of the pathogenic microorganisms in the subgingival microenvironment is indicative of a successful periodontal therapy [4,5].

The adjunctive use of antimicrobial agents in addition to non-surgical therapy has shown to provide additional benefits [6]. The use of systemic antimicrobial agents in the treatment of periodontal disease was widespread in the past. The main drawback of systemic antimicrobials is achieving and maintaining a therapeutic concentration at the infected site. Antimicrobial agents locally applied into the periodontal pockets may further suppress periodontal pathogens. Various local drug delivery (LDD) systems have been introduced to overcome the disadvantages of the systemic route of antimicrobial administration. LDD systems in periodontal therapy can provide up to 100-fold higher drug doses at the target site compared to systemic administration [7]. To date, doxycycline, and metronidazole are the most widely used antibiotics for LDD in the treatment of periodontal disease. The concentration of tetracycline in gingival crevicular fluid (GCF) is 5–10-fold higher than serum levels due to their anti-collagenase property [8,9]. Such antimicrobial therapy as an adjunct requires reduced dosage and fewer applications and also has high patient compliance.

Increasing concern over the unwanted side effects and emergence of highly resistant microbes with increased pathogenicity at the treated sites has altered the general perception of the capabilities of these antimicrobial agents [10,11]. In light of this, there is a need to look for alternate options that are effective, relatively safe, and economical [12]. Research in phytosciences, an emerging multidisciplinary science, has revealed various medicinal plants possessing antimicrobial activity with fewer side effects, reduced toxicity, and cost-effectiveness. Extracts of these medicinal plants offer a new choice for optional antimicrobial therapy against various oral microorganisms [13,14].

Keeping the above factors in mind, the present study was planned with the aim to assess and compare the effectiveness of locally administered 2% gel made from lemongrass polymer and 10% doxycycline hyclate gel as an adjunct to SRP in treating chronic periodontitis and also to verify and compare the antimicrobial effect of 2% gel made from lemongrass polymer and 10% doxycycline hyclate gel in chronic periodontitis.

## 2. Materials and Methods

### 2.1. Study Design

A double-blind parallel arm randomized controlled study was planned to evaluate the effectiveness of 2% lemongrass gel as an adjunct to SRP therapy in chronic periodontitis with 10% doxycycline hyclate gel as active control. This study also aimed to evaluate in vivo antimicrobial effect of both LDD agents. The study protocol was developed, and all subjects gave their informed consent for inclusion before they participated in the study. The study was conducted in accordance with the Declaration of Helsinki, and the protocol was approved by the Ethics Committee of Institute of dental science, Bareilly, India. “(IDS/ETHCC/14/10).” The study was registered with the Clinical Trial Registry of India (CTRI REF/2021/03/042330 AU).

Study subjects and Sample size

Sample size (*n*) was calculated by using formula

(1) $$\;n=\frac{2{\left(Standard\;deviation\right)}^{2}}{{\left(Effect\;size\right)}^{2}}{\left(Z_{\alpha /2}+Z_{1-\beta }\right)}^{2}$$

where Z_α/2_ = 1.96, Z_1−*β*_ = 0.842 are respectively the 95% confidence value obtained from the standard normal distribution with power of the study at 80%. At least seventeen subjects were needed to detect a significant difference in PPD after intervention with an effect size of 0.50 and a standard deviation of 0.45 from the pilot study using ten subjects. To compensate for the dropouts, 10% of *n* is added to get the final sample size. Hence, the final minimum sample size in each group was *n* = 20.

A total of 74 subjects were screened for the study, of which 34 subjects did not satisfy the inclusion criteria. The inclusion criteria for the patient selection were: (a) patients in the age group of 18–60 years, (b) untreated chronic periodontitis with a minimum of four periodontal pockets per quadrant, and (c) periodontal pockets with probing depth 4 to 6 mm. Patients with systemic diseases, who had received antibiotics during the previous three months, or who had received antiseptic/antiplaque agents in the last three months, pregnant women, and patients who had undergone periodontal treatment in the last six months were excluded from the study. The details of study procedure were explained to all eligible subjects (*n* = 40) and consent was obtained.

### 2.2. Formulation of 2% Lemongrass Gel and 10% Doxycycline Hyclate Gel

For this study, a stable bio-absorbable controlled-release formulation of 2% lemongrass gel and 10% doxycycline hyclate for the treatment of periodontal pockets was prepared by Department of Pharmacy, Mahatma Jyotiba Phule Rohilkhand University, Bareilly, India [20].

#### 2.2.1. Preparation of 2% Lemongrass Gel

Appropriate quantity of Carbopol 934 was soaked in water for a period of 2 h. Carbopol was then neutralized with Triethanolamine (TEA) by stirring. Then, 2% lemongrass essential oil was dissolved in appropriate and pre-weighted amounts of propylene glycol and ethanol. The solvent blend was transferred to the Carbopol container and agitated for an additional 20 min. The dispersion was then allowed to hydrate and swell for 60 mins, then the pH was adjusted with 98% TEA until the desired pH value approximately reached (6.8–7). During pH adjustment, the mixture was stirred gently with a spatula until a homogeneous gel was formed.

#### 2.2.2. Preparation of 10% Doxycycline Hyclate Gel

A total of 5% sesame oil was added to 95% of the melted Glyceryl monooleate (GMO) at 60–70 °C with continuous stirring. After the above solution was cooled to room temperature, 10% of doxycycline hyclate was added to it until a homogenous gel was obtained.

### 2.3. Study Procedure

**Figure 1:**
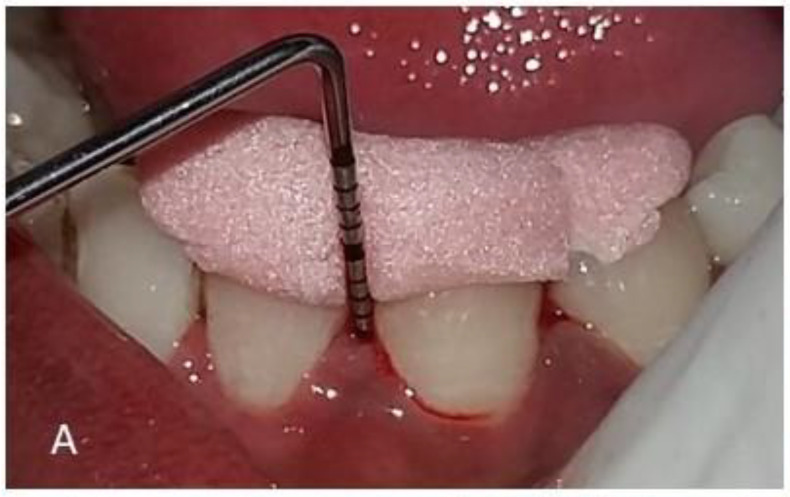

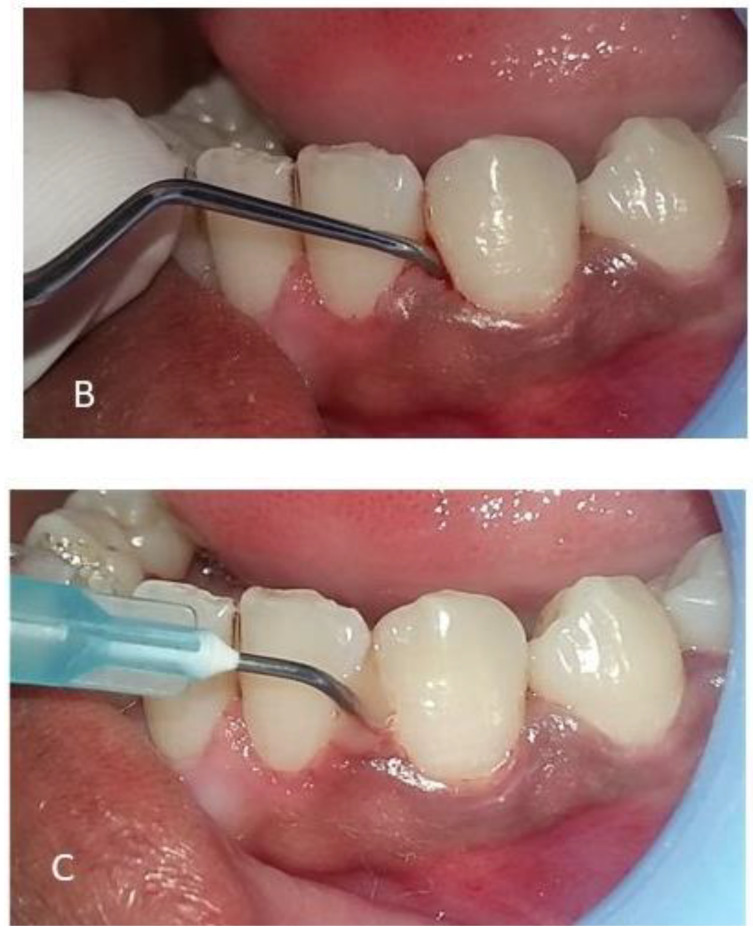

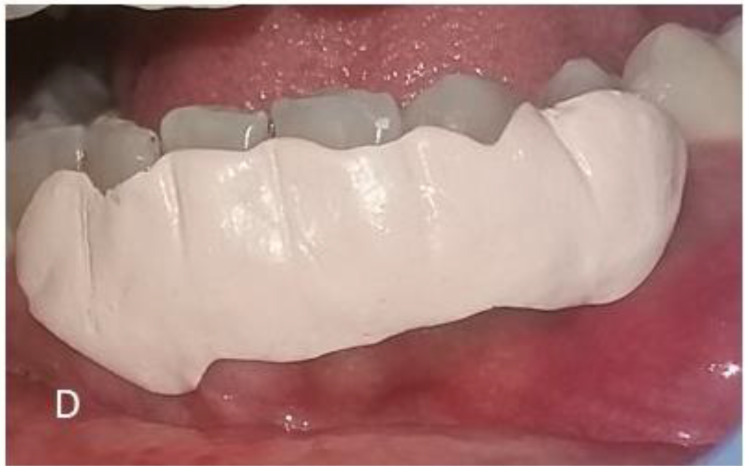
Clinical steps involved in the study. (**A**) Recording clinical parameters at baseline. (**B**) Subgingival plaque sample collection. (**C**) LDD at the site of Periodontal Pocket. (**D**) Placement of periodontal dressing.

Forty subjects were randomly allocated into two equal intervention groups using a lottery method with Group A subjects (*n* = 20) for 2% lemongrass gel and Group B (*n* = 20) for 10% doxycycline hyclate gel as interventions, respectively. Only one investigator was aware of the intervention groups, and the subjects were coded for identification. The same investigator carried out the placement of local drug delivery for each study subject. The clinical periodontal status of each subject was recorded at baseline, i.e., before SRP therapy and application of local drug delivery and on 1st and 3rd month follow-up assessments. The clinical steps involved are shown in Figure 1.

In this study both clinical and microbiological periodontal parameters were used for outcomes assessment.

### 2.4. Clinical Assessments

Clinical assessments for periodontitis were done as per American Academy of Periodontology (AAP) 1999 classification [21]. The indices used were: Gingival index (GI), Plaque Index (PI) Probing Pocket Depth (PPD) and Clinical Attachment Level (CAL) [1,22,23]. All clinical assessments were done with the University of North Carolina-15 probe (Hu Friedy^®^). A single blinded investigator was trained and calibrated for recording clinical indices with ten independent subjects on an interval of 24 h. The intra-class correlation coefficient was found to be 0.82 which indicate good reliability.

### 2.5. Microbiological Analysis

Before recording clinical parameters, subgingival plaque samples were collected from the selected sites at baseline, 1st month and 3rd month visits to evaluate the changes in colony forming units (CFU) of primary periodontal pathogens, i.e., *Porphyromonas gingivalis*, *Actinomyces naeslundii* and *Prevotella intermedia* [24]. The teeth were isolated using cotton rolls and a plaque sample was obtained using sterile area specific Gracey curettes (Hu Friedy^®^) in a previously fumigated minor operation theatre. The samples were then transferred in a vial containing 10 mL Robertson Cooked Meat Broth Medium and were incubated at 37 °C for 24–96 h. The total number of CFU’s was determined based on serial dilution from 10–1 to 10–3 on selective media. Finally, each bacteria’s count was determined based on typical colony and bacterial morphology in 10^2^ CFU/milliliter on Muller Hinton Agar.

### 2.6. Study Intervention

**Figure 2:**
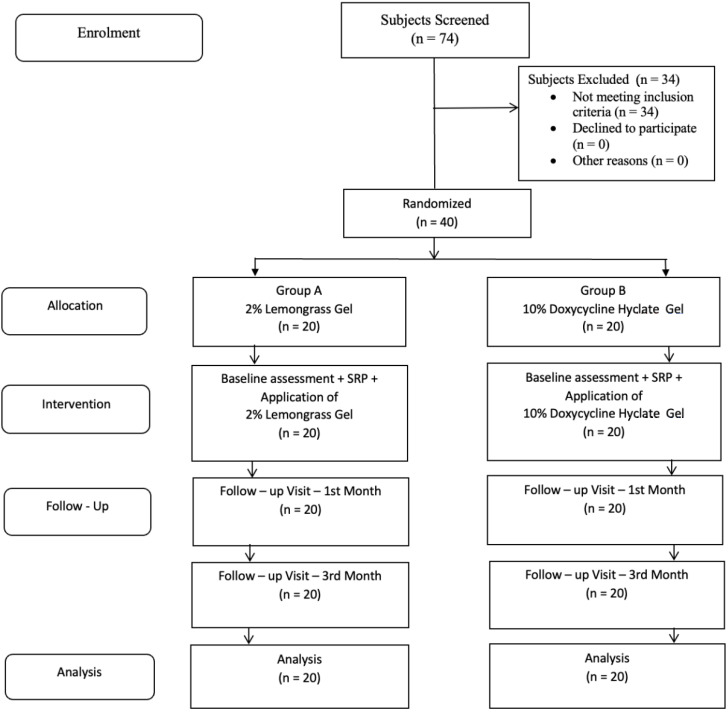
Flow chart of the study.

Immediately after baseline assessment, all subjects received SRP therapy followed by application of local drug delivery. The investigator assigned for application of LDD gels applied 2% lemongrass gel and 10% doxycycline hyclate gel into periodontal pockets as per patient codes. The LDD gel was inserted into the bases of the pockets using a special syringe with a blunt cannula. The end of the blunt cannula was moved coronally to fill the pocket, and the excess gel was removed using a curette or wet cotton pellet. The site was covered with a periodontal dressing (Coe-Pak^®^) to prevent the medication from being flushed out of the pocket. All subjects were advised to use 0.2% of chlorhexidine rinses, and standard oral hygiene instructions were given. After one week, the subjects were reviewed for any discomfort such as transient discomfort, erythema, transient resistance, allergy following treatment, and visual examination to record any soft tissue changes after removal of periodontal dressing. The subjects were recalled for follow-up assessments on 1st and 3rd months, and oral hygiene maintenance was reinforced. The study design flow chart is given in Figure 2. All subjects who participated completed the study, and none of the subjects had any complications during recall visits.

### 2.7. Statistical Analysis

The collected data was first entered into MS-Excel spreadsheet and further subjected to analysis using SPSS for Windows, Version 16.0. Chicago, SPSS Inc. (Chicago, IL, USA) Descriptive results were presented as frequency, mean, and standard deviation. Inter-group comparisons were made by independent Student’s *t*-test and intra-group comparisons to test changes during follow-up visits were obtained by using repeated measures of ANOVA test and Least Significant Difference post hoc test. The level of significance was set at 5%.

## 3. Results

At baseline, there was no statistically significant difference between the groups in their mean clinical and microbiological scores (*p* > 0.05) (Table 1). The repeated measures ANOVA test showed that there was a significant reduction in all clinical mean scores from baseline to the 1st and 3rd month follow-ups in both 2% lemongrass gel and 10% doxycycline gel groups (*p* < 0.05) (Table 2). Similarly, there was significant reduction in all microbiological mean CFU scores from baseline to the 1st and 3rd month follow-ups in both 2% lemongrass gel and 10% doxycycline gel groups (*p* < 0.05) (Table 3). A pairwise Least Significant Difference (LSD) post hoc test showed that there was a significant reduction of GI, PPD, CAL, and all three microbiological mean CFU count scores in both the 2% lemongrass gel and 10% doxycycline gel groups from baseline to the 1st and 3rd month follow-up scores (*p* < 0.05). There was no statistically significant difference in the mean PI scores from baseline to the 1st and 3rd month follow-up scores in 2% lemongrass gel group (*p* > 0.05) (Table 2 and Table 3).

The mean difference was highest in PPD and CAL assessments from baseline to the follow-up visits in both groups. The mean differences of microbial CFUs from baseline to the 1st and 3rd month follow-ups in both groups were comparable (Table 2 and Table 3).

## 4. Discussion

Various polymers have been explained by researchers for drug delivery. Different polymers exhibit different mucoadhesive properties depending on their physical and chemical strength. For example, a more flexible polymer exhibits a higher degree of mucoadhesive property [25]. Mucoadhesive polymers possessing hydrophilic functional groups such as COOH, OH, NH_2_, and SO_4_H are more favorable candidates for the formulation of targeted drug delivery. These polymers bearing the desired functional group interact with mucus through physical entanglement as well as through chemical bonds resulting in the formation of a cross-linked network. For example, urea is a well-accepted hydrogen-bonding disruptor that decreases the mucoadhesion of mucin/pectin samples. Other properties that may affect the mucoadhesive nature of the polymer include chain length, degree of hydration, degree of cross-linking, polymer concentration, charge, etc.

In the last three decades, periodontal therapy has seen significant progress in various aspects. There has been a shift from surgical treatment procedures to techniques and methods aimed at delivering the drug locally along with scaling and root planing to the affected sites by targeting the specific periodontopathic microorganisms in bringing improvements in clinical parameters of the periodontium. The adjunctive use of antimicrobial agents in addition to non-surgical therapy has been shown to provide additional benefits. Such antimicrobial therapy as an adjunct needs a reduced dosage and fewer applications and should also have high patient compliance. Various LDD systems have been introduced to overcome the disadvantages of the systemic route of antimicrobial administration. A number of LDD systems have been used in different clinical trials with different degrees of success. Upon analysis of the various clinical reports, it is seen that most of the LDD systems have resulted in significant improvement in the clinical parameters. However, the kind of improvement in the clinical parameters has not been consistent. Many of the published research on LDD systems in the literature has not evaluated the change in the microbial count of periodontopathic bacteria.

In the present research, we have used Carbopol 934 for the formulation of 2% lemongrass gel, which eventually yielded many therapeutic benefits by releasing the drug in a sustained manner. Carbopol or carbomer are high molecular weight polymers of acrylic acid cross-linked with either allyl sucrose or allyl ethers of penta erythritol. These contain 56% and 68% of carboxylic acid groups calculated on the dry bases [26]. These are used as suspending agents or viscosity-increasing agents, dry and wet binders, as well as rate-controlling agents in tablets, enzyme inhibitors of intestinal protease in peptide-containing dosage forms, etc. Carbomer is a pH-dependent polymer that stays in solution form at acidic pH but forms a low viscosity gel at alkaline pH. Carbopol offers the advantage of exhibiting excellent mucoadhesive properties in comparison with other polymers (e.g., cellulose derivatives and polyvinyl alcohol) [27]. Different mucoadhesive formulations containing carbopol have been developed and it was found that these demonstrated excellent mucoadhesive properties and release the drug in a controlled manner for a longer period of time. Tan et al. [28] developed a bioadhesive gel incorporating lidocaine using carbopol and Polyvinylpyrrolidone (PVP). The results indicated that an increase in carbopol concentration significantly increased gel compressibility, hardness, and adhesiveness, that is, the factors that affect the ease of gel removal from the container, ease of gel application onto the mucosal membrane, and gel bioadhesion, respectively. Moreover, the resulting formulation provided a sustained release as compared with the conventional dosage forms. Similar results were obtained by Bilensoy et al. [29]. They developed 5-FU containing thermosensitive, mucoadhesive gel based on carbopol 934 and pluronic F12 for the treatment of Human Papillomavirus (HPV)-induced cervical cancer. The resulting formulation demonstrated better anticancer activity at lower doses, avoiding unwanted side effects of the drug. In another study, Patel and Chavda [30] prepared amoxicillin-loaded gastroretentive microspheres using carbopol-934 providing sustained release.

In the present study, there was significant reduction in all clinical mean scores from baseline to 1st and 3rd month follow-up in both 2% lemongrass gel and 10% Doxycycline Gel groups. Similarly, there was significant reduction in all microbiological mean CFU scores from baseline to 1st and 3rd month follow-up in both 2% Lemongrass Gel and 10% Doxycycline Gel groups. The mean differences for both groups from baseline to 1st and 3rd month follow-up scores showed that there was significant reduction of GI, PPD, CAL and all three microbiological mean CFU count scores in both 2% Lemongrass Gel and 10% Doxycycline Gel groups. The mean difference was highest in PPD and CAL assessments from baseline to follow-up visits in both groups. The mean differences of microbial CFU’s from baseline to 1st and 3rd month follow-up in both groups were comparable.

This is attributable to the antibacterial property of doxycycline and lemongrass. Studies have shown that the beneficial effects of doxycycline in periodontal diseases are due to the antibacterial property of doxycycline against periodontopathogens [6] and the inhibitory action of the pathologically elevated tissue degrading activities of matrix metalloproteinases (MMPs) in the inflamed gingival tissues of adult periodontitis [9]. The control group-associated results of the present study are in accordance with the findings of the previous studies [6,24].

Lemongrass has several beneficial properties that can be of use in periodontal therapy. Previous studies have demonstrated the anti-inflammatory and antimicrobial properties of lemongrass in terms of inhibition of the production of interleukine-1β (IL-1β) and IL-6 [31]. Citral, the main component of the lemongrass, is responsible for its anti-microbial property by causing extensive leakage of critical molecules and ions from the bacterial cell and permeabilization of the bacterial cytoplasmic membrane leading to their death [32] and in addition to this, the anti-inflammatory action of lemongrass is due to the blockage of the LPS-induced activation of Nuclear Factor kappa-B (NF-ĸB) [33,34]. The observations of the test group in the present study are in accordance with the findings of previous studies [17,35,36]. In addition to this, lemongrass oil has also been shown to decrease the volatile sulfur compounds, hence inhibiting halitosis [36].

There was no statistically significant difference in mean PI scores from baseline to the 1st and 3rd month follow-up scores in the 2% lemongrass gel group. This could be because of a lack of oral hygiene maintenance.

The results of this study suggested that 2% lemongrass gel as an LDD system is one of the nonsurgical treatment modalities in bringing improvement in clinical & microbiological parameters. However, further investigation with a larger sample size on a prolonged post-operative follow-up is required to conclusively establish the effectiveness of this gel.

## 5. Conclusions

It could be concluded that the local delivery of 2% lemongrass gel as an adjunct to scaling and root planing is effective and comparable to 10% doxycycline gel in the treatment of chronic periodontitis, and 2% lemongrass gel is an effective antimicrobial agent against primary periodontal pathogens, i.e., *Porphyromonas gingivalis*, *Actinomyces naeslundii,* and *Prevotella intermedia*. Further studies targeting other periodontal pathogens associated with specific periodontal conditions need to be conducted.

## Figures and Tables

**Table 1:** Comparison of mean scores of both clinical and microbiological parameters at baseline.

	Groups	N	Mean	SD	*p*-Value
GI	2% Lemongrass Gel	20	1.78	0.26	0.868
	10% Doxycycline Gel	20	1.76	0.24	
PI	2% Lemongrass Gel	20	1.82	0.20	0.814
	10% Doxycycline Gel	20	1.84	0.19	
PPD	2% Lemongrass Gel	20	5.30	0.47	0.744
	10% Doxycycline Gel	20	5.35	0.49	
CAL	2% Lemongrass Gel	20	5.50	0.51	0.759
	10% Doxycycline Gel	20	5.55	0.51	
Porphyromonas Gingivalis (10^3^ CFU)	2% Lemongrass Gel	20	1.06	0.54	0.757
	10% Doxycycline Gel	20	1.00	0.60	
Actinomyces Naeslundi (10^2^ CFU)	2% Lemongrass Gel	20	0.69	0.43	0.683
	10% Doxycycline Gel	20	0.75	0.51	
Prevotella Intermedia (10^3^ CFU)	2% Lemongrass Gel	20	1.12	0.48	0.274
	10% Doxycycline Gel	20	0.94	0.52	

Statistically significant at 5% level of significance.

**Table 2:** Comparison of mean clinical parameters scores within groups at baseline, 1 month, and 3 month follow-ups.

	Groups	Assessment Period	Mean	SD	*p*-Value
GI	2% Lemongrass Gel	Baseline	1.78	0.26	<0.0001 * Ф
		1 Month	1.34	0.27	
		3 Months	1.22	0.28	
	10% Doxycycline Gel	Baseline	1.76	0.24	<0.0001 * Ф
		1 Month	1.34	0.23	
		3 Months	1.16	0.28	
PI	2% Lemongrass Gel	Baseline	1.82	0.20	0.021 *
		1 Month	1.78	0.25	
		3 Months	1.73	0.23	
	10% Doxycycline Gel	Baseline	1.84	0.19	0.015 * Ф
		1 Month	1.72	0.26	
		3 Months	1.70	0.28	
PPD	2% Lemongrass Gel	Baseline	5.30	0.47	<0.0001 * Ф
		1 Month	3.30	0.47	
		3 Months	3.25	0.44	
	10% Doxycycline Gel	Baseline	5.35	0.49	<0.0001 * Ф
		1 Month	3.40	0.50	
		3 Months	3.15	0.37	
CAL	2% Lemongrass Gel	Baseline	5.50	0.51	<0.0001 * Ф
		1 Month	3.50	0.61	
		3 Months	3.45	0.60	
	10% Doxycycline Gel	Baseline	5.55	0.51	<0.0001 * Ф
		1 Month	3.60	0.50	
		3 Months	3.35	0.49	

* Statistically significant at 5% level of significance by repeated measures ANOVA. Ф Statistically significant at 5% level of significance by pairwise Least Significant Difference post hoc test.

**Table 3:** Comparison of mean microbiological parameter scores within groups at baseline, 1 month, and 3 month follow-ups.

	Groups	Assessment Period	Mean	SD	*p*-Value
Porphyromonas Gingivalis (10^3^ CFU)	2% Lemongrass Gel	Baseline	1.06	0.54	<0.0001 * Ф
		1 Month	0.31	0.34	
		3 Months	0.28	0.31	
	10% Doxycycline Gel	Baseline	1.00	0.60	<0.0001 * Ф
		1 Month	0.37	0.33	
		3 Months	0.38	0.35	
Actinomyces Naeslundi (10^2^ CFU)	2% Lemongrass Gel	Baseline	0.69	0.43	<0.0001 * Ф
		1 Month	0.33	0.34	
		3 Months	0.31	0.30	
	10% Doxycycline Gel	Baseline	0.75	0.51	<0.0001 * Ф
		1 Month	0.33	0.31	
		3 Months	0.32	0.31	
Prevotella Intermedia (10^3^ CFU)	2% Lemongrass Gel	Baseline	1.12	0.48	<0.0001 * Ф
		1 Month	0.70	0.36	
		3 Months	0.67	0.35	
	10% Doxycycline Gel	Baseline	0.94	0.52	<0.0001 * Ф
		1 Month	0.51	0.41	
		3 Months	0.52	0.41	

* Statistically significant at 5% level of significance by repeated measures ANOVA. Ф Statistically significant at 5% level of significance by pairwise Least Significant Difference post hoc test.

## Data Availability

Data can be made available on demand by the chief researcher for academic purposes by email.
